# Navigating trauma: Venezuelan women's and adolescent's experiences before and after migration amidst the humanitarian crisis

**DOI:** 10.1016/j.jmh.2024.100299

**Published:** 2025-01-01

**Authors:** C. Correa-Salazar, J.J. Amon, K.R. Page, A.K. Groves, E.N. Agudelo-Avellaneda, D.S. Torres-Benítez, A. Martínez-Donate

**Affiliations:** a Psychology Department, Universidad de los Andes, Bogotá, Colombia; b Johns Hopkins Medicine, Baltimore, United States; c Community Health and Prevention Department, Drexel University, Philadelphia, United States

**Keywords:** Women, Adolescents, Migration, Humanitarian crisis, Colombia, Risks, Health

## Abstract

Migrant and refugee women and adolescents are extremely vulnerable in humanitarian crisis and armed conflict contexts. The Venezuelan crisis has unleashed the largest exodus of migrants/refugees in recent Latin American history, most of whom have relocated to Colombia. There is a scarcity of research addressing the how adverse and traumatic experiences related to violence presents mental health amidst the Venezuelan-Colombian humanitarian crisis context and how it affects communities in relocation communities. This study sought to explore how traumatic experiences pre-, during and post-migration might relate to mental health risks for Venezuelan migrant and refugee women and adolescents and assess feasible mechanisms that can protect and promote these populations upon relocation in Colombia. We conducted semi-structured interviews with 30 migrant women and adolescents, inclusive of cis- and transgender women in two Colombian cities using a participatory-action research framework. Key findngs suggest that parental abandonment in origin contexts, experiences of household and community violence before and after migration and structural barriers to access services are main factors impacting this population. Feasible strategies to promote and support wellbeing and better mental health access ought to include community leaderships and community-based support networks post-migration that can support trust in services, disseminate information and engage vulnerable groups in services.

## Highlights

• Women and adolescents are especially vulnerable during humanitarian and armed conflict contexts where there is often a lack of legal and institutional protections, and significant disruption of protective structures like family units and support networks.
• There are important evidence gaps and methodological challenges in measuring gender-based violence and adverse childhood experiences in humanitarian crises settings where fear, stigma, lack of reporting mechanisms, and norms of secrecy make data collection challenging in household and community settings.
• Key findings suggest parental abandonment in origin contexts, experiences of household and community violence before and after migration and structural barriers to access services are main risk factors impacting for the mental health of women and adolescents amidst the venezuela-colombia humanitarian context pre-, during and post-migration.
• Feasible strategies to promote and support mental health and wellbeing for women and adolescents in relocation contexts in colombia ought to include community leaderships and community-based support networks post-migration that can support trust in services, disseminate information and engage vulnerable groups in needed mental health services.

## 1 Introduction

Children, adolescents and women are especially vulnerable during humanitarian (Ruiz and Rodríguez, 2020; CEPAZ, 2018) and armed conflict contexts (Stark and Landis, 2016; Bendavid et al., 2021; Wirtz et al., 2014) where there is often a lack of legal and institutional protections, and significant disruption of protective structures like family units and support networks (Bendavid et al., 2021; Stark and Ager, 2011). Transit and relocation phases can increase risks of physical and sexual violence, human trafficking, kidnapping, economic hardship, and child labor which especially impact these populations (C. Correa-Salazar et al., 2023). Lack of economic resources and food insecurity in humanitarian emergencies can force them into sexual exploitation, servitude marriages, and transactional sex, which overall increases health risks and violates human rights (Rodríguez-Lizarralde et al., 2022 Aug; Gongora Arias, 2022).

Unaccompanied migrant/refugee children and adolescents and those left behind report higher rates of having suffered physical violence, sexual abuse, and witnessing abuse to others when compared to non-migrant children and migrant adults (McLeigh, 2013; Duque-Páramo, 2012; Jud et al., 2020). Migration also interrupts educative processes, which impacts the availability of social and protective networks. These experiences can impair their physical, emotional, and social development (Stark and Landis, 2016), resulting in long-lasting harms (Stark and Landis, 2016) that can be amplified by the limited availability of services (Temin et al., 2013). Evidence from this and other contexts has shown increased risk of suicide, depression, anxiety, post-traumatic stress (PTSD), alcohol and substance use, and emotion dysregulation as consequences of armed conflict, social violence and migraton on children and adolescents’ mental health (Bendavid et al., 2021; McLeigh, 2013; Duque-Páramo, 2012; Jud et al., 2020; Schmidt, 2022). These have also been conceptualized as adverse childhood experiences (ACEs) (Boullier and Blair, 2018; Flaherty et al., 2013)

Research on migration has also found a strong correlation between female gender and violence, with women and girls reporting higher rates of sexual violence, psychological and physical abuse and neglect amidst humanitarian crises and migration contexts (Schmidt, 2022). Gender-based violence (GBV) is especially prevalent among the reproductive age groups ((Schmidt, 2022-49) (Wirtz et al., 2014; Calderón-Jaramillo et al., 2020). When family structures are broken up, girls are often charged with care labor at home, hindering their opportunities and development (Ayala-Carrillo M del and Cárcamo-Toalá, 2012). Adolescents have been identified as an important at-risk group, but little research focuses on this population (C. Correa-Salazar et al., 2023). Rates of GBV against women and adolescents, inclusive of cis- and transgender women, are high in complex emergencies, and prevention responses remain important priorities (Stark and Ager, 2011). There are important evidence gaps and methodological challenges in measuring rates of GBV in humanitarian crises settings where fear, stigma, lack of reporting mechanisms, and norms of secrecy make data collection challenging (Stark and Ager, 2011). There is also a lack of evidence on how GBV and traumatic experiences in migration contexts present risks to mental health within the home and community environments after relocation (Stark and Ager, 2011). Relocated populations are understood in this study as groups and individuals who choose to resettle and rebuild their lives in Colombia, but continue to self-identify as migrants/refugees from Venezuela.

Venezuela is the most violent country in Latin America (World Population Review, 2022) and one of the most violent in the world (García Pinzón and Mantilla, 2021). Colombia hosts almost 3 million Venezuelan migrants and refugees, being the main recipient of Venezuelan forcibly-displaced populations (The United Nations Refugee Agency [UNHCR], 2022). This crisis has collided with the “post” Colombian armed conflict, creating a context of cross border risk factors for already vulnerable migrants (Human Rights Watch, 2020), who face increased violence and barriers to access care upon relocation due to lack of a legal migratory status, discrimination, disinformation and conservative views about sexual and reproductive health normalized in both cultures (C. Correa-Salazar et al., 2023; Calderón-Jaramillo et al., 2020; Soto et al., 2021). The post-armed conflict context impacts the migratory phenomenon through armed actors and guerrilla groups’ control of illegal border crossings between both countries, human trafficking networks, forced recruitment into armed groups and forced labour, corruption and overall social violence along the border, in migration routes and relocation contexts (Human Rights Watch 2020; C. Correa-Salazar et al., 2023).

Despite robust humanitarian efforts to address the crisis, little focus has been given to how traumatic experiences (including adverse child experiences -ACEs- and GBV) during various stages of migration (including pre) can impact mental health and coping strategies for vulnerable populations. Traumatic experiences significantly increase the risk of adverse mental health outcomes, albeit heterogeneity in how people respond to trauma. To date, there is little examination of the mental health risks that these experiences might represent for the migrant and refugee population despite prioritization by humanitarian actors (UNICEF, 2021; Bell et al., 2012), especially for vulnerable groups like adolescents, women and transgender individuals relocated in Colombia. This scarcity of research hinders prevention efforts that could be enacted to protect mental health outcomes like increased anxiety, depression and post-traumatic stress disorder, ensure equity in care and promote interventions beyond urgent health needs (Stark and Landis, 2016). This study aimed to explore how traumatic experiences pre-, during and post-migration might relate to mental health risks for Venezuelan migrant and refugee women and adolescents. We also sought to assess feasible mechanisms that can protect and promote these populations upon relocation in Colombia.

## 2 Methods

This study was part of a larger four-year participatory action-research (PAR) project based on a university-community partnership implemented between 2019 and 2023 focused on understanding health disparities affecting Venezuelan migrant and refugee women and girls’ health and identifying sustainable structures of care (C. Correa-Salazar et al., 2023, 2023). All fieldwork was conducted in Colombia in February-July 2021 in the two cities that account for the largest populations of relocated migrants: Bogotá and Cúcuta (Migración Colombia, 2021). We also implemented eleven sexual and reproductive health (SRH) workhops with 230 migrant and refugee women and adolescents in the two study sites to respond to community needs following participatory methodology.

We conducted thirty semi-structured interviews with migrant/refugee women and girls (Kvale, 2008) in the two study sites following a constructivist grounded theory (CGT) methodology (Charmaz, 2014). Participants responded to a sociodemographic questionnaire first. Each interview lasted between 45 and 90 min. The semi-structured interview guide was built upon extensive formative research and validated with peer researchers. It included violent experiences across the lifecourse, migratory experiences and perceived health risks (Heise et al., 2019) during migration phases of pre-departure, transit, border crossing and relocation (Zimmerman et al., 2011). Questions related to adolescent and childhood experiences focused on family dynamics, relationships with close relatives, caretakers and community members, origin contexts, perceived challenges and adverse child experiences and support networks. All research activities were done in Spanish and conducted by the P.I. (CCS) and a local team of researchers and peer leaders. We followed a systematic inductive, iterative, and comparative approach to explore risks and violence experienced by adult and adolescent particpants pre- and post- migration.

### 2.1 Participants

Thirty Venezuelan migrant and refugee self-identified women and adolescents (*n* = 30) participated in the interviews analyzed in this study (see Table 1 for demographics). We implemented a non-probabilistic purposive sampling methodology (Lincoln and Denzin, 2000) using local partner's contact networks to support trust (Faugier and Sargeant, 1997). Criteria for participation included being Venezuelan, self-identifying as women, being between 14 and 49 years of age, being relocated to Colombia and migrating through terrestrial means. Emancipation criteria for adolescents sampled was fulfilled by having migrated alone and lacking adult guardians in Colombia. Emancipation was confirmed through community partners. We used emancipation as an inclusion criteria to sample adolescents that had migrated alone or with peers and who were identified by our local partners and extant literature as an at-risk group falling through the cracks of the humanitarian response: not qualifying as children or families, not having any migratory papers and not wanting to access services for fear of deportation or stigmatization. Albeit hard-to-reach, emancipated adolescents ended up making 30 % of the sample and consistently attended participatory health workshops. Half of participants lived in Cúcuta and half in Bogotá. These cities are key transit and destination sites, but also add between both the largest numbers of relocated migrants/refugees in Colombia.

**Table 1:** Socio-demographic information on participants.

Demographics		N	%	Cúcuta	Bogotá
**Age**					
	14–18	6	20 %	2	4
	19–23	7	23 %	2	5
	24–28	6	20 %	3	3
	29–33	4	13 %	4	0
	34–43	2	7 %	1	1
	44–49	5	17 %	3	2
**Gender identity**					
	Cisgender	24	80 %	15	9
	Transgender	6	20 %	0	6
**Sexual orientation**					
	Heterosexual	28	94 %	15	13
	Lesbian	1	3 %	0	1
	Other	1	3 %	0	1
**Race/ethnicity**					
	Mixed race	26	87 %	13	13
	Black	4	13 %	2	2
**Occupation**					
	Transactional sex	13	44 %	5	8
	Informal work	10	33 %	7	3
	Student	3	10 %	1	2
	Unemployed	4	13 %	2	2
**Was abandoned by a parent or caretaker**		20	67 %	10	10
**Has survived sexual violence**		20	67 %	10	10

### 2.2 Data analysis

All interviews were audio recorded and transcribed verbatim by research assistants and an external transcription service. All interviews were conducted, transcribed, and coded in Spanish by three independent coders (including the P.I.). The analysis was performed using Charmaz's (Bell et al., 2012) grounded theory approach (Charmaz, 2014). The analysis was done after all data was collected.

We held weekly discussions as we read and explored interviews. Each transcript was read, re-read, and coded line by line in an initial coding phase (Charmaz, 2014, 2006). We concluded line-by-line coding once the most salient codes were identified. We continued with a focused coding phase to examine and expand initial codes (Charmaz, 2014). Finally, we organized tentative analytic categories through an axial coding phase that established relationships between categories and subcategories (Saldaña, 2015). To focus the analysis on childhood and adolescent experiences, we centered our discussions and main themes around what participants recounted as lived experiences when being young, the experiences of their children and if they were underage, their current conditions. The analysis was undertaken using word and excel processors. Discrepancies were solved through discussions during weekly meetings and consultations with local partners and community peers involved in the project. Axial codes integrated emergent themes, and memos in Dedoose®. We confirmed and expanded findings in community gatherings. Selected quotes were translated into English by the P.I. (CCS).

### 2.3 Ethics

The Institutional Review Board of Drexel University (protocol #2009,008,067) provided ethics approval and a reliance agreement was established with the Colombian Ministry of Health and Social Protection to conduct research locally. All participants provided verbal informed consent after discussing anonymization of results, termination of interviews at any time and receiving contact information to obtain results and resolve questions. Our local partner handled referrals to local child/health/justice institutions. Both the IRB approval and the reliance agreement approved the use of oral consent for all participants to protect anonymity. Oral consents and assents were documented for in audio in Spanish. The IRB approved these conditions before the start of the research. We baed our approach in participatory care ethics, promoting socially responsible actions through the re-defining of subjects as active participants instead of vulnerable subjects (Cahill, 2013). Engaging emancipated adolescents (*n* = 6) was an important part of our research to ensure a truly inclusive perspective of migrating girls’ realities and needs. All participants were informed of their rights and were offered a $15 USD incentive and a transportation subsidy to compensate their time. We also provided referrals to psychological care, medical evaluations and medicines as requested. To ensure protection of participants, research staff involved in data collection activities underwent a 2-month training covering study aims, ethical procedures for research on sensitive topics, referral pathways, GBV and child protection praxis, use of audio-equipment, and qualitative research methods. Research staff were under close supervision by the P.I., a licensed psychologist. De-identified data was stored on a secure server.

## 3 Results

Participants were all Venezuelan between 14 and 49 years old (reproductive age). Most identified as cisgender, and 20 % identified as transgender. Sixteen were, or had been, teenage mothers, 66 % had experienced parental abandonment as children or adolescents and 66 % were survivors of sexual violence. Almost half engaged in transactional sex at the time of data collection and 70 % were involved in some form of informal work. Table 1 shows demographics for participants. Quotes refer to participants’ experiences as children/adolescents or current experiences in the case of emancipated adolescents.

In analyzing interview data, three themes emerged around adverse childhood experiences (ACEs) and potentially traumatic experiences of migrants pre- and post-departure experienced as children and adolescents: a) parental abandonment and lack of support structures before migration, b) household and community violence continuum before and after migration, and c) structural barriers to access resources after relocation to Colombia. Conversely, we found two emerging themes around mechanisms to address the protection of children and adolescent mental health: a) positive aspects related to migration; and b) community peer leaderships. Building on previous work from the Crossroads project (C. Correa-Salazar et al., 2023, 2023, 2023), we conceptualize emerging theories within a cross-border risk environment that encompasses structural determinants of health (C. Correa-Salazar et al., 2023) encompassing pre-migration contexts, transit and relocation cities (C. Correa-Salazar et al., 2023).

### 3.1 Perceived adverse and traumatic experiences

#### 3.1.1 Parental abandonment and lack of support structures before migration

Participants discussed neglect and abandonment by parents and partners as a big part of experienced traumatic events as children and for their children: “I had no dad, I lived with mom. She's a Christian and a cook. My kids have no dad, I'm their mom and dad” (Cúcuta, 27 years old). Family separations and absence of a parental figure that provides protection and material conditions for safety were common among participants’ accounts of their early years and the lives of their own children. Twenty participants described suffering some form of parental abandonment.

Forced-migration caused by the humanitarian crisis in Venezuela also induced separation of families, increasing risks for children due to disintegration of support structures and the lack of the necessary conditions for children to migrate with parents. A participant explains: “She tells me: ‘mom, I want to go with you and work where you are’… But, how do I explain? The place where I live is very bad, it's not private and to bring them… If I go to work… What can I do? They're all female [her children]. I can't, I can't have them with me” (Cúcuta, 31 years old). Even for those who had a stable family structure prior to leaving Venezuela, migration and separation were perceived as potential risks for mental health later on or upon relocation to Colombia. Parental abandonment and neglect were also related by participants to child labour, lack of food in the household and exposure to violence by family and community members.

#### 3.1.2 Household and community violence continuum

When discussing childhood's and adolescents’-specific mental health risks related to GBV and ACEs, participants expressed having survived different forms of violence in family and community environments. The most common forms of violence perpetrated against them were sexual violence at the hands of someone close, witnessing intra-familial violence between parents or within families, and directly experiencing or witnessing community violence related to threats, extorsion, fights, murders and physical attacks in origin and relocation contexts.

Three participants described having suffered sexual abuse from a family member during their early childhood in Venezuela (2–7 years old). Abuse was related to parental absence and exposure to community violence. A participant explains: “…we washed our clothes in the river… everyone had a mom and a dad but I didn't… so I think that's one of reasons why that man did what he did [sexual abuse], because I had no one to look after me.” (Cúcuta, 24 years). Sexual violence and abuse created a risk environment within the household. Sixteen participants had been or were teenage mothers and twenty-one witnessed and experienced intra-familial violence at home before migration, factors that where often closely related in interviews:

My daughter's father hit me, that's why I say I didn't have a happy childhood, or happy adolescence. Because I was a teenage mom, I focused on protecting my daughter… my dad hated me because I am female, then a man abuses me as a girl and finally my partner hits me, kicks me, takes out my eye almost… when I am a teenager. I had no childhood, no adolescence…… (Cúcuta, 21 years old).

Six participants reported being involved in child labor and four had been sexually exploited before the age of fifteen (including two trans women), mostly by parents and close relatives: “…my family had worked [in the street] to earn money quick and easy. My cousins, my aunts… they took me, told me where to stand and what to say” (Cúcuta, 17 years old). Even though these dynamcs started pre-migration, they continued after relocation to Colombia.

Transgender participants recounted being expelled from family homes in Venezuela, and finding in transational sex the only means to survive. They described being exposed to physical violence and threats as a consequence of these situations: “…my dad wouldn't take me as I am, I had to feed myself, so I forced myself into prostitution… I was 9 years old and I cried endlessly after….” (Bogotá, 21 years old). Household expulsion and interpersonal violence within households was a key driver for migration for transgender women and related to physical and sexual abuse, depressive symptoms or suicidal conducts: “I always searched for ways on how to hang myself, kill myself. Because all the things I lived with my family… since I was little. You know?” (Bogotá, 19 years old).

Sexual, interpersonal and community violence during childhood, adolescent years and in the adult age occurred oftentimes before migrating, causing push factors for migration and for separation from families. However, after migration, sexual violence risks and physical abuse continued. For participants, children in relocation contexts continued to be exposed to risks similar to those of origin contexts (i.e. transactional sex, sexual abuse, and substance abuse in public spaces). A participant describes: “…the same people who rent, they see a pretty girl and the mom that can't pay and they say, ‘give me your daughter for 15 days’, and moms have to give in or else they have to sleep in the street with their other children” (Cúcuta, 27 years old).

Community violence was also experienced as a pervasive factor impacting children and adolescents’ wellbeing and increasing exposure to traumatic experiences before and after migration. Most participants came from neighborhoods with reported extorsions, presence of gangs and physical threats and had to relocate to neighborhoods that were perceived as dangerous due to drug trafficking dynamics, armed groups’ control, high levels of prostitution and trafficking networks: “… I don't let my children out to play. I'm always watching them. I have heard the stories of children being abducted. There are several who have been disappeared… no one has seen them since. Police don't listen because we're migrants” (Bogotá, 37 years old). A third of participants (both adults and emancipated adolescents) explicitly mentioned fearing children's abduction in relocation contexts and recounted personally knowing or having witnessed kidnappings. Emancipated adolescents further declared being afraid themselves of being kidnapped or raped by armed actors. Thus, children's movements are restricted in relocation contexts as they bare witness to interpersonal and community violence. They are also recipients of mothers’ and caregivers’ coping mechanisms to deal with trauma experienced before and after migration in a continuum of lifecourse violence: “…I become someone else, some days I act on the frustration I feel and yell at my daughter… Lord, forgive me, help me take in all the things I've to experience…” (Cúcuta, 24 years old).

**Structural barriers to resources after relocation to Colombia.** Participants perceived that experiencing barriers to services like education, housing, healthcare and child welfare after relocation to Colombia impacted their health and increased risks. For emancipated adolescents who migrated as a consequence of family expulsion, violence or searching for sexual and reproductive services to care for pregnancies, education barriers endured due to irregular migratory status, lack of identification documents and lack of information on the education system where identified as main problems to ensure a livelihood. A participant explains: “I'm not in school because I got pregnant, and I need to buy my daughter's things. Nobody's gonna give me that” (Cúcuta, 15 years old). Another participant described: “My kids are not in school because they told us we needed the migratory permit. They've been out of school for a year.” (Cúcuta, 19 years old).

Healthcare barriers to access mental health services and/or sexual and reproductive care were also common for children and adolescents post-migration. Lacking a legal migration status and identification documents, being stigmatized for being a teenage mother, having more than one child, lacking a stable job and being perceived as a sex worker were all factors related to healthcare barriers and information for adolescents in Colombia. Teenage participants and adults that migrated as minors expressed fear of hospitals contacting child services and losing custody of their children: “…when my baby got sick the second time, they told me that if I ever took him there again, they would call child services [Bienestar Familiar]. I decided never to take him to the hospital again… because I was underage.” (Cúcuta, 23 years old). For underage mothers, the fear of child services involved not only losing custody of their children but their own freedom and livelihoods. But they also fear being abused. A participant explains: “…I'm afraid they'll take me. They took my baby brother… child services picked him up and then he escaped because they were beating him, making him sleep on the floor… what if they did that to me or my children?” (Cúcuta, 16 years old).

In Colombia, child services, police and institutional agents were perceived as victimizers by migrant women and adolescents. Participants expressed being chased and extorted by police when engaging in transactional sex or being perceived as such, being robbed of their documents and money and asked for sexual favors in exchange for freedom by police and migration agents. Being underage was an additional barrier to protect themselves because their claims are not taken seriously by police and/or institutions, and they are threatened with child services referrals. A participant describes: “A guy harassed me… and I went to the police, and they defended him, not me. They told me: ‘you're Venezuelan, you have no right to be here, you better thank us for letting you go as a minor…’ So, I left.” (Cúcuta, 17 years old). Another explains: “the police told me ‘how much money have you got…?… I started crying, I explained it was for my son… but they stripped me and sexually assaulted me.” (Cúcuta, 21 years old).

### 3.2 Mechanisms for protecting migrant women, adolescents and children

#### 3.2.1 Positive aspects related to migration

Despite suffering violence, discrimination and barriers in relocation contexts, participants described multiple positive aspects for their life and for coping with trauma, dealing with the consequences of adverse childhood experiences and violence derived from the migratory experience. A key theme was the possibility to access food, mental health and reproductive. health services, medicines and support from community and/or civil society organizations. A participant describes: “… for the medicines, I took my child to the Red Cross and she's underweight, they gave her what she needed… now for my ‘special child’ [child with a disability], I took him too and they gave him the medicines he needed” (Cúcuta, 45 years old).

Support systems after relocation were often community-based, led by religious or humanitarian actors. Through these networks, participants reported being able to find the mental health services they needed: “…a woman that works with me told me where I could find psychological help. I went there [to a local NGO] and told them I had troubles with my daughter and trouble with myself and I needed help. I found my therapist there and I'm still going…” (Bogotá, 25 years old). However, these services often lacked sustainability given limited eight-session protocols or high rotation of providers in services. Bogotá’s services were perceived as more accessible, acceptable and sustainiable, whereas Cúcuta's were perceived as scarce and stigmatizing by participants.

#### 3.2.2 Peer leadership

A main theme that participants perceived related to improving their life conditions and coping with traumatic experiences and violence was support networks around community leaders and peers. In Colombia, many migrant/refugee women and girls are trained as community leaders by humanitarian and civil society organizations. This outreach strategy bridges information gaps and breaks stigma barriers associated to services, improving access to mental health and sexual and reproductive health providers, peer leaders and networks of support that help build trust between services and communities. A participant explains:

…there are many girls without a dad and a mom, no one to look after them and tell them what's what. They have a bad… guidance, you know? Why do I take it on myself to guide them? Because they look for help to take care of their kids, or to get condoms, or a pregnancy test, or an HIV test. How do I become a leader? Because my daughter ran away and in searching for her, I started to encounter all these stories of sadness. So if I see that me and my husband can contribute something to all these girls, a guidance, a service, a mom figure or a spiritual one.. we do it. (Cúcuta, 28 years old).

Peer leadership often starts with beneficiaries of services. Then, community outreach strategies engage women and girls interested in becoming peer leaders and provide free trainings on different skills, service provision pathways, and referral networks. Participants who had been trained or who had accessed these services, reported being trained as a peer leader serves to re-signify past-violent experiences, migratory grief, and trauma suffered before, during or after migration. For women and adolescents, peer networks help deal with the impacts of parental abandonment and family separation, barriers to education and sexual and physical abuses suffered in household and community environments. Peer leaders ultimately help build trust back into communities.

## 4 Discussion

This research aimed to explore how traumatic experiences pre-, during and post-migration might relate to mental health risks for Venezuelan migrant and refugee women and adolescents and assess feasible mechanisms that can protect and promote their health and access to protective services upon relocation in Colombia, particularly to Cúcuta and Bogotá. Key themes expose how parental abandonment and lack of support structures before migration, household and community violence in pre- and post-migration contexts and structural barriers to access services upon relocation are major factors impacting health and wellbeing for these structurally vulnerable groups. Furthermore, these factors present obstacles to their inclusion and participation in relocation contexts as they continue to suffer, witness and experience the intergenerational consequences of trauma and social violence. Emancipated adolescents, teenage mothers and women are stigmatized by their age, migration status, life choices, perceived identities and family compositions, which imposes barriers to access protective services in relocation contexts. Conversely, protective structures and positive aspects derived from migration relate to being able to access peer-led support networks, mental health services, and healthcare not available in origin contexts. Migration was also perceived as a way to find new life purposes in supporting others and resignifying negative traumatic experiences.

For participants, lack of legal and institutional protections pre-, during and post-migration were significant obstacles to access mental health services and protective structures, which aligns with available evidence amidst complex emergencies (Stark and Ager, 2011). We also found transit and relocation phases as potentially increased risk environments for physical and sexual violence, human trafficking, and economic hardship (C. Correa-Salazar et al., 2023) due to armed actors’ control of borderlands, precarity in migratory conditions and lack of resources like food or jobs. These conditions increase risks for mental health upon relocation. Findings enforce the need to consider how these violent and difficult contexts can victimize emancipated minors and women in humanitarian emergencies (Rodríguez-Lizarralde et al., 2022; Gongora Arias, 2022).

We also found that experiences of violence for participants before, during and after migration were profoundly gendered. Research on migration has found a strong relation between being female and violence, with women and girls reporting higher rates of sexual violence, psychological and physical abuse and neglect Results support these findings and show a high prevalence of GBV and ACEs among participants, connecting these experiences to perceived risks to mental health within the home and community environments in origin and relocation contexts.

This research contributes valuable evidence on the impacts of household and community violence on women and adolescents amidst humanitarian crisis and South-to-South pre-, during and post-migration contexts (Stark and Landis, 2016), and on the detrimental impacts these conditions can have on relocated communities in Colombia (Stark et al., 2023). Our findings suggest that to effectively protect these groups amidst this complex humanitarian emergencies like the cross-border context that encompasses the Venezuelan crisis, there is a need to strengthen country-level processes and accountability mechanisms that address household violence and reporting mechanisms -including sexual abuse of minors, parental abandonment and neglect, and child exploitation (Stark and Ager, 2011). Results also point to the importance on addressing patriarchal values that shape unequal gender relationships. In both origin and relocation contexts, large numbers of cases go unreported due to lack of information on reporting mechanisms, and the fact that violence is often perpetrated by parents, relatives, acquaintances or institutions charged with their protection (police, child services, hospitals) (Stark and Ager, 2011). Accordng to our findings, reporting teams working in tandem with community leaderships are feasible approaches to bridge the gap between information and justice access in relocation contexts. In Colombia, the integration of services of GBV and child protection should be aligned to address protection needs, as drivers of violence are largely interconnected across public and private domains and exacerbated in migration and relocation contexts (Bermudez et al., 2019) where families are separated or there is parental abandonment. Surveillance and community-based mechanisms to report the disappearance of minors should also be put in place through community leaderships to prevent kidnappings and abuses. These feasible strategies could help bridge the gap between vulnerable populations and found needs that can decrease the risks to mental health in relocation contexts in Colombia.

In this regard, continued collaborations between government, humanitarian and civil society actors is pivotal to strengthen data systems. Government-humanitarian actors’ platforms have been working successfully towards integration and protection efforts (like the Interagency R4 V group) and the Gender-based violence information management systems (GBVIMS), which was specifically designed to coordinate GBV cases across agencies (GBVIMS, 2024). However, sustainability and high rotation of personnel challenge efforts to engage relocated and in-transit groups (Ruiz and Rodríguez, 2020). This causes a lack of follow-up on cases, disaggregation of information systems and neglect in protection mechanisms (McLeigh, 2013). Efficient coordination across all levels, as well as integration of information systems (including the education system that has the most complete record of migrant children and adolescents in Colombia) could provide additional support for migrant families’ transition into relocation, (Puga, 2023) strengthen reporting mechanisms and address mental health determinants in community settings (Villegas et al., 2021). Succesfull policies like the Temporal Statute of Protection (ETPV, according to the acronym in Spanish) that increased regularization and inclusion in work and education sectors for migrants are feasible and reachable tools to support these action and information systems. Education inclusion is an effective way to mitigate drivers of violence as it supports protective environments like schools and teachers’ supervision. This infrastructure is also helpful when disseminating trainings on positive parenting, emotional regulation strategies and relationship skill-building for families, among other relevant information for migrants.

Succesful policies like the ETPV, could be built on to promote inclusion in education, the labour market and housing for emancipated adolescents and teenage mothers (McLeigh, 2013). Availability of housing for emancipated minors and their children is paramount, as well as eliminating migration permits as a requirement to access healthcare, which is difficult for unaccompanied minors without documents. Institutional agents like police, and medical doctors should be included in campaigns aimed at eliminating gender stereotypes and information barriers to accessing rights, permitting the engagement of migrants in referral routes, protection mechanisms and access to justice linkages -including presenting claims for police abuse and brutality for emancipated minors (Amnistía Internacional [Amnisty International], 2022). Children welfare systems should not interfere with access to healthcare, mental health services and sexual and reproductive health for teenage mothers, especially where there is a lack of transparent reporting mechanisms as discussed by participants. Joint work with civil society organizations and peer leaders could facilitate responding to women's and specific-adolescents’ needs, follow-up on cases, promote inclusion and build trust in relocation communities from a gender and age-perspective aligned with human rights frameworks (McLeigh, 2013).

To address community-level factors that place women and adolescents at risk of maltreatment, neglect, and intra-familial violence (Stark and Landis, 2016), there is a need to address intersecting drivers of violence that have roots in hierarchical gender norms and to develop initiatives that consider intergenerational mental health inequities. Participants discussed having suffered ACEs and GBV across the lifecourse, which might represent mental health risks for them and their children (Sangalang and Vang, 2017; Chou and Buchanan, 2021). Specific community-based actions to improve mental health access could be the inclusion of peer leaders in outreaching strategies, services and government initiatives. Peer-led models of care have been successful for engagement of at-risk populations in this and other contexts (Greene et al., 2022). Platforms like United Nations High Commissioner for Refugees (UNHCR) and the International Organization for Migrations (IOM) have successfully done this in the studied cities in Colombia to engage hard-to-reach migrant groups in services and improve mental health access. Actions supporting the sustainability of these collaborations could help address intergenerational drivers of violence that stem from negative or violent experiences in caretakers’ early childhood, or that related to humanitarian crisis factors or stress-related conditions during migration (Sangalang and Vang, 2017) As described in this study, accessing available and acceptable mental and other health services in different moments of the migratory journey is beneficial to deal with past and present trauma.

This study presents some limitations. Sampling was done through community partners, so findings are subject to response bias. Given the qualitative participatory design, this study might not reflect experiences of migrants before and after the data collection period or outside of our sampled networks and does not reflect the experiences of boys and men. We chose to focus solely on women and adolescent girls given emerging and pervasive evidence that amidst this particular emergency, they were facing increased sexual, physical and social violence. However, the ethics of care procedures that informed consent processes, data collection, and analysis should have reduced some of these biases. Given that participants were relocated in Bogotá and Cúcuta, findings cannot be interpreted as common across Colombian cities or Latin America. Bogotá and Cúcuta represent different groups of migrants and different migration contexts that might affect how participants experience migration and migration contexts. Still, these cities include the largest numbers of relocated migrants in Colombia, and encompass a diverse group of experiences. Findings can also be altered by the COVID-19 pandemic, which could have mitigated some biases in terms of travelling options and access to healthcare. To address this, we covered a wide enough period to include diverse migration experiences. Another limitation refers to recall errors for adult participants, given that we inquired about traumatic events experienced as children and adolescents. However, these were compared and analyzed in conjunction with participant adolescent's present experiences to deepen the understanding of multiple traumatic experiences and ACEs over the lifecourse.

## 5 Conclusion

Our study contributes valuable evidence for those working with migrant/refugee children and adolescents in the region. Particularly, it addresses evidence gaps on how traumatic experiences (including ACEs and GBV) during various stages of migration (including pre) impact migrants and refugee women and adolescents and might present mental health risks in relocation contexts. Our discussion of the experiences that impact women and adolescents, and might act as push factors for migration, contributes to understand and address drivers of risk, GBV and ACEs in both origin and relocation contexts where fear, stigma, lack of reporting mechanisms, and institutional victimization make data collection challenging (Stark and Landis, 2016; Stark and Ager, 2011).

## Funding

This research was funded by the Dissertation Award of the Urban Health Collaborative at 10.13039/100008211Drexel University to conduct a pilot project on urban health. Grant # 284,081–6905.

## CRediT authorship contribution statement

**C. Correa-Salazar:** Writing – review & editing, Writing – original draft, Validation, Supervision, Project administration, Methodology, Investigation, Funding acquisition, Formal analysis, Data curation, Conceptualization. **J.J. Amon:** Writing – review & editing, Validation, Supervision, Conceptualization. **K.R. Page:** Writing – review & editing, Supervision, Conceptualization. **A.K. Groves:** Writing – review & editing, Conceptualization. **E.N. Agudelo-Avellaneda:** Formal analysis, Data curation. **D.S. Torres-Benítez:** Formal analysis, Data curation. **A. Martínez-Donate:** Writing – review & editing, Writing – original draft, Validation, Methodology, Conceptualization.

## Declaration of competing interest

The authors declare that they have no known competing financial interests or personal relationships that could have appeared to influence the work reported in this paper.
